# Biological age acceleration in Alzheimer’s disease modulates relative cortical to medial temporal lobe neurodegeneration

**DOI:** 10.1016/j.neurobiolaging.2025.06.003

**Published:** 2025-06-04

**Authors:** Lasya P. Sreepada, Christopher A. Brown, Sandhitsu R. Das, Paul A. Yushkevich, David A. Wolk, Corey T. McMillan

**Affiliations:** a Department of Bioengineering, School of Engineering and Applied Sciences, University of Pennsylvania, Philadelphia, PA 19104, United States; b Department of Neurology, Perelman School of Medicine, University of Pennsylvania, Philadelphia, PA 19104, United States; c Department of Radiology, Perelman School of Medicine, University of Pennsylvania, Philadelphia, PA 19104, United States

**Keywords:** Epigenetics, Alzheimer’s disease, DNA methylation, Biological aging, MRI, Neurodegeneration

## Abstract

Alzheimer’s disease (AD) is highly associated with aging, typically presenting with amnestic, multi-domain cognitive impairment and greater medial temporal lobe (MTL) atrophy relative to cortex. However, approximately 15 % of AD cases present atypically, often at younger ages and with greater cortical involvement relative to MTL. This association between age and AD presentation is imperfect: some younger-onset cases are typical, amnestic presentations while some older-onset cases present less typically. We hypothesize that this discordance may be partially modulated by discordance between chronological age and biological age, defined epigenetically. Participants from the Alzheimer’s Disease Neuroimaging Initiative with MRI and known amyloid status were selected (*n* = 1011, 44.4 % female, 75.33 ± 7.28 years) and classified as amyloid-negative, cognitively unimpaired (*n* = 329) or amyloid-positive, symptomatic individuals with mild cognitive impairment or dementia (*n* = 682). Biological age was estimated in individuals with DNA methylation (*n* = 448) using established epigenetic clocks. Biological age gap (BAG) was calculated to categorize individuals into “accelerated” (biological age > chronological age) or “decelerated” (biological age < chronological age) groups. We define the Cortico-Medial Temporal index (CoMeT), derived from MRI, to quantify age-adjusted relative differences between cortical and MTL structures. Lower CoMeT scores indicate relatively greater cortical involvement. BAG and CoMeT were significantly correlated (Pearson *R*=0.13, *p* = 0.023). Symptomatic individuals with decelerated BAG exhibited significantly lower CoMeT scores than individuals with accelerated BAG, with a large effect size, reflecting greater cortical involvement relative to MTL (Wilcoxon *p* = 0.023, rank-biserial correlation=−0.98). We conclude that biological aging modulates AD presentation beyond chronological age, providing novel insights into mechanisms underlying AD heterogeneity.

## 1. Introduction

Alzheimer’s disease (AD) is an age-related neurodegenerative condition, and there is increasing recognition of the extensive heterogeneity in its clinical presentation. While clinical AD typically presents after 65 years of age, approximately 15 % of individuals with AD present atypically and between 5 % and 10 % (Sirkis et al., 2022) have a younger age of onset (Koedam et al., 2010; Graff-Radford et al., 2021; Sirkis et al., 2022). AD is most often considered an amnestic, multi-domain disorder with memory loss most prominent due to early accumulation of tau and neurodegeneration in the medial temporal lobe (MTL). However, other less common phenotypes are known to occur, including predominant language, visuospatial, or executive impairments that are often referred to as cortical-predominant presentations of AD, due to disproportionate atrophy in cortical regions relative to the MTL (Patterson et al., 2000; Murray et al., 2011; Dickerson et al., 2017; Ferreira et al., 2017; Graff-Radford et al., 2021; Polsinelli and Apostolova, 2022). Although it is the case that these presentations occur more frequently in individuals with early onset of disease, the contributions of aging mechanisms to AD heterogeneity are still poorly understood.

While chronological age is a known driver of AD pathogenesis (Brookmeyer et al., 1998; Swerdlow, 2007), there has been a surge in studies of *biological* aging in the past decade. There is particular interest in developing markers of biological aging using the epigenome or other “omics” data that are highly dynamic over the lifespan (Horvath, 2013; Levine et al., 2018).

While biological age has been quantified using various methods and data modalities (Jylhävä et al., 2017), recent statistical approaches have focused on machine learning algorithms trained to predict age, mortality risk, or other age-associated phenotypes from DNA methylation profiles, termed epigenetic clocks (Hannum et al., 2013; Horvath, 2013; Levine et al., 2018; Lu et al., 2019; Belsky et al., 2022). Further, these epigenetic clocks have been used to measure the discordance between chronological age and biological age, known as the “biological age gap” (BAG), which represents the degree of age acceleration (biological age > chronological age) or deceleration (biological age < chronological age) in an individual (Salih et al., 2023). As such, two individuals with the same chronological age may show phenotypic differences that could be explained by differences in their BAGs. For instance, accelerated biological age has been associated with many age-related outcomes, including mortality risk, frailty, and neurodegenerative conditions such as AD (Chen et al., 2016; Jain et al., 2022; Gao et al., 2024). Consistent with standard nomenclature, we adopt the terms “individuals with accelerated BAG” and “individuals with decelerated BAG” to describe groups who are estimated to be biologically older or younger on average than their chronological age, respectively.

**Fig. 1.**
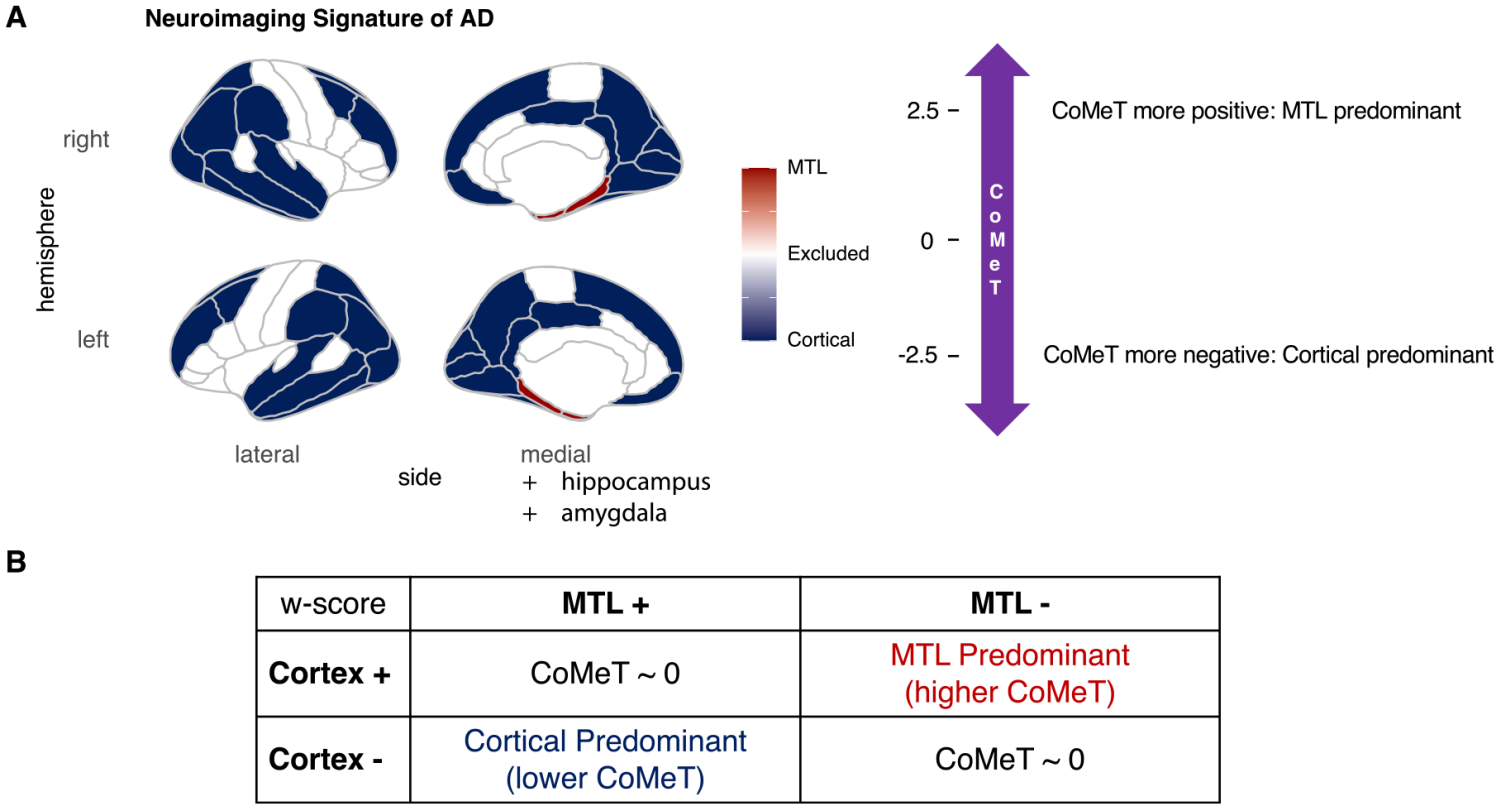
The Cortico-Medial Temporal index (CoMeT). This study assesses the spectrum of neurodegenerative pattern in AD, which varies along a continuum from cortical-predominant to limbic-predominant (MTL focused). We define CoMeT to quantify the relative involvement of cortex to MTL. (A) Brain map (left) providing a visualization of the bilateral DKT atlas ROIs considered as part of cortex (blue): inferior temporal, temporal pole, superior parietal, precuneus, supramarginal, superior frontal, middle frontal, fusiform, inferior parietal, superior temporal, posterior cingulate, isthmus cingulate, lateral occipital, middle temporal, medial orbitofrontal, cuneus, lingual, and pericalcarine regions; and MTL (red): entorhinal cortex and parahippocampal gyrus. Volumes of hippocampus and amygdala were also included in the MTL. Schematic diagram (right) providing interpretation of CoMeT, which quantifies relative cortical and MTL atrophy. (B) Table providing interpretation of CoMeT. Lower values of CoMeT indicate greater cortical involvement relative to MTL and correspond to less typical presentation. Conversely, higher values of CoMeT indicate greater MTL involvement relative to cortex, corresponding to more typical presentation. AD = Alzheimer’s disease; DKT = Desikan-Killiany-Tourville; MTL = Medial Temporal Lobe. ROI = Region of Interest.

In this paper, we evaluate the potential contributions of biological aging, defined by DNA methylation, to AD heterogeneity. We hypothesize that age-related biological factors are the mechanism by which chronological age modulates AD presentation, thus we predict that biological age acceleration modulates atypicality in AD beyond chronological age alone. Given the evidence that less typical neurodegenerative patterns in AD are associated with earlier disease onset (Koedam et al., 2010; Graff-Radford et al., 2021; Sirkis et al., 2022), this study tests the *a priori* hypothesis that, for a given chronological age, individuals with decelerated BAG (i.e., biologically younger) are likely to have less typical, cortically predominant presentations and conversely that individuals with accelerated BAG (i.e., biologically older) are likely to have more typical, limbic-predominant presentations. To investigate this, we developed the **Co**rtico-**Me**dial **T**emporal index (CoMeT): a novel score of disease atypicality in AD that is based on relative cortical to MTL involvement (Fig 1). CoMeT is a continuous measure derived from *in vivo* structural MRI and compares structures of signature brain regions involved in AD, which were previously established by neuroimaging studies (Bakkour et al., 2009; Dickerson et al., 2009; Möller et al., 2013; Touroutoglou et al., 2023). Specifically, CoMeT is defined as the signed difference of cortical thickness in non-MTL signature regions and of thickness or volume in MTL regions. Lower values of CoMeT indicate relative cortical predominance (i.e., less typical presentation), while higher values indicate relative MTL predominance (i.e., more typical presentation). We apply established epigenetic clocks (Horvath, 2013; Hannum et al., 2013; Shireby et al., 2020) to DNA methylation profiles to estimate biological age and BAG in symptomatic individuals. After adjusting for chronological age, we test whether CoMeT is significantly lower in individuals with decelerated BAG compared to those with accelerated BAG. In other words, we expect biological aging to influence the neurodegenerative pattern beyond the known effect of chronological age. By exploring the interplay between biological aging and disease presentation, we aim to deepen our understanding of the biological mechanisms underlying heterogeneity and atypicality in AD.

## 2. Materials and methods

### 2.1. Participants and study design

We included 1011 individuals from the Alzheimer’s Disease Neuroimaging Initiative (ADNI) (Petersen et al., 2010; Weiner et al., 2017) who had an MRI scan and known amyloid status. Amyloid status was determined using the nearest PET or CSF measure within 2 years of baseline MRI, which we defined as the earliest timepoint with the smallest interval between MRI and DNA methylation, or simply the earliest timepoint for participants who did not have DNA methylation. We defined amyloid positivity using previously defined PET standardized uptake value ratios (SUVR) cut-offs for florbetapir (^18^F-AV45 SUVR ≥ 1.1) or Pittsburgh Compound B (PiB SUVR ≥ 1.47) (Landau et al., 2013), when available, or CSF β-amyloid peptide (Aβ-42 < 980) otherwise (Hansson et al., 2019).

**Fig. 2.**
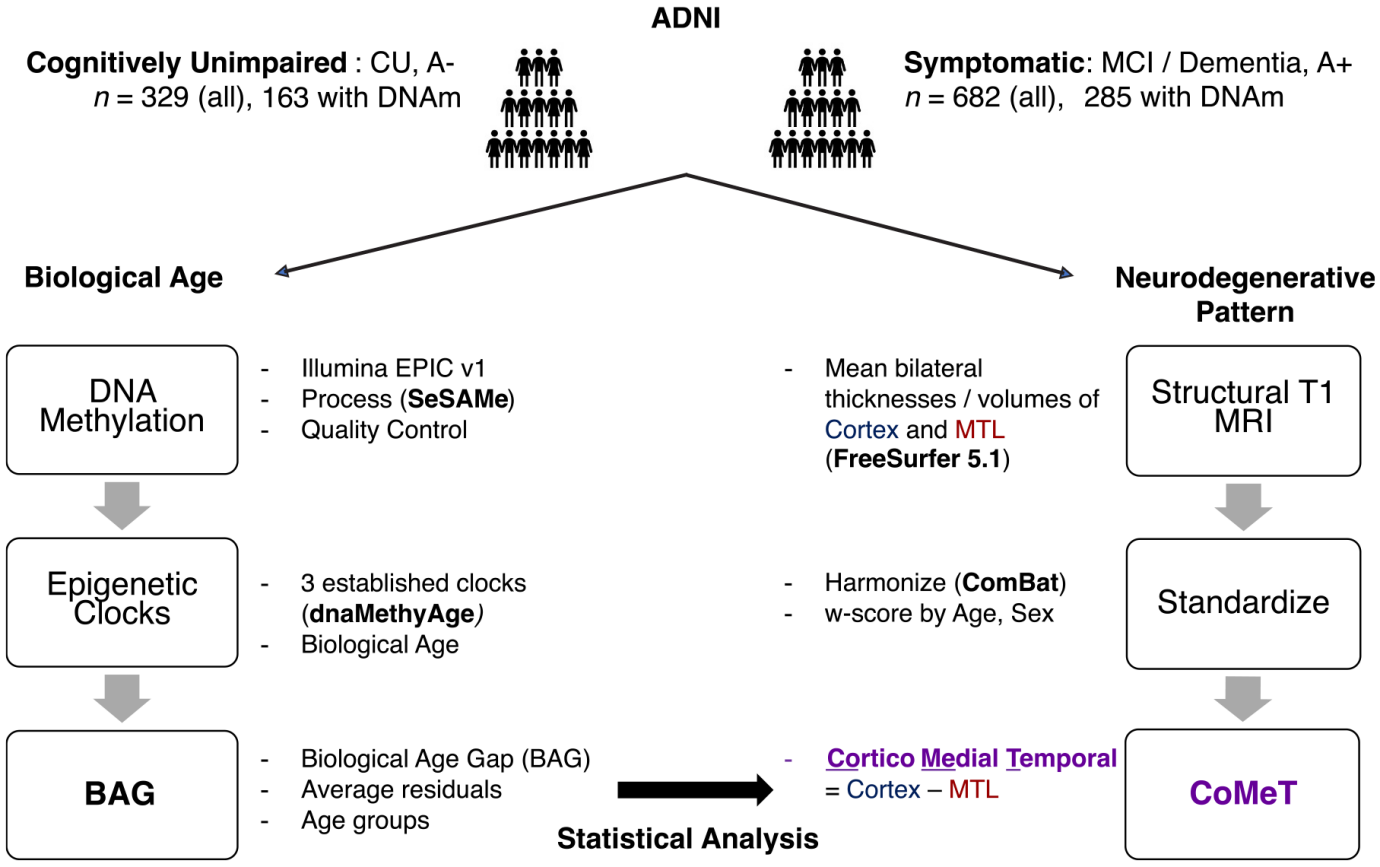
Schematic diagram of the study design. This is a retrospective, cross-sectional case-control study. Participants from ADNI with MRI and amyloid status were selected, a subset of which also had whole-blood DNA methylation data. Participants were matched for age, sex, and education level and defined as CU individuals, who were amyloid-negative, or symptomatic individuals with MCI or dementia due to AD, who were amyloid-positive. Biological age was estimated using established epigenetic clocks for participants with DNA methylation. Age acceleration was computed for each clock age by regressing biological age against chronological age and extracting residuals. To mitigate noise from individual epigenetic clocks, residuals were averaged across clocks, giving the biological age gap (BAG) used throughout the study. We define CoMeT, an index to quantify relative cortical to MTL involvement along a continuum, using regional thickness measures from *in vivo* structural MRI. Wilcoxon rank-sum tests and Pearson correlation were used for statistical analysis of the association between BAG and CoMeT. A+ /− = amyloid positive/negative; AD = Alzheimer’s disease; ADNI = Alzheimer’s Disease Neuroimaging Initiative; CU = Cognitively Unimpaired; CoMeT = Cortico-Medial Temporal index; MCI = Mild Cognitive Impairment.

Participants were further divided into two groups based on clinical consensus diagnosis closest to baseline MRI: cognitively unimpaired (CU) individuals (*n* = 329) who are amyloid-negative, and symptomatic individuals with either mild cognitive impairment (MCI) or dementia due to AD (i.e., amyloid positive, *n* = 682). Among these individuals, a subset of 448 individuals had DNA methylation data (Vasanthakumar et al., 2020), which was required for analyses of biological aging, including 163 CU individuals and 285 symptomatic individuals. A schematic diagram of the cross-sectional study design and inclusion/exclusion criteria is provided in Fig 2. The Clinical Dementia Rating scale Sum-of-Boxes (CDRSB) (Morris, 1993; O’Bryant et al., 2008) was used as a measure of global function and cognition.

### 2.2. Biological age markers using epigenetic clocks

DNA methylation data from whole blood were available in ADNI for 448 individuals who satisfied the study criteria as described above. DNA methylation was assayed using the Illumina Infinium Human Methylation EPIC v1.0 BeadChip (Pidsley et al., 2016), which assesses methylation levels at over 850,000 phosphorylated cytosine-guanine dinucleotide (CpG) sites across the epigenome. Raw intensity files were processed using the *SeSAMe* R package (Zhou et al., 2018) according to standard protocols, including sample filtering, CpG probe filtering, signal correction, and normalization. Quality control procedures ensured at least 95 % signal detection at each CpG, and Uniform Manifold Approximation and Projection (UMAP) (McInnes et al., 2018) was used to check for sample separation by covariates (see Figure S1).

Three established epigenetic clocks (Horvath, 2013; Hannum et al., 2013; Shireby et al., 2020) were applied to processed DNA methylation data to compute biological age and BAG. The *dnaMethyAge* R package (Wang et al., 2024) was used to compute biological ages and the regression-based BAGs using the clocks referenced above. We opted for the regression-based measure because it reduces systematic deviations in BAG across datasets (Dhingra et al., 2018). There were no adjustments for covariates in the BAG calculation, as they were accounted for in downstream analyses. For timepoints with technical replicates, one replicate was randomly selected for further analysis.

While there is inherent noise in epigenetic clock measures due to variation in the populations and tissues used to train epigenetic clocks (Shireby et al., 2020), there is also high covariance amongst clock measures. To mitigate noise from sample variance, we averaged BAGs from the Horvath, Hannum, and Shireby clocks (Horvath, 2013; Hannum et al., 2013; Shireby et al., 2020). This average is referred to simply as “BAG” throughout the rest of the paper and is the measure used in analyses. We report sensitivity analyses within individual clocks in Figure S2.

#### 2.2.1. Biological age groups

**Fig. 3.**
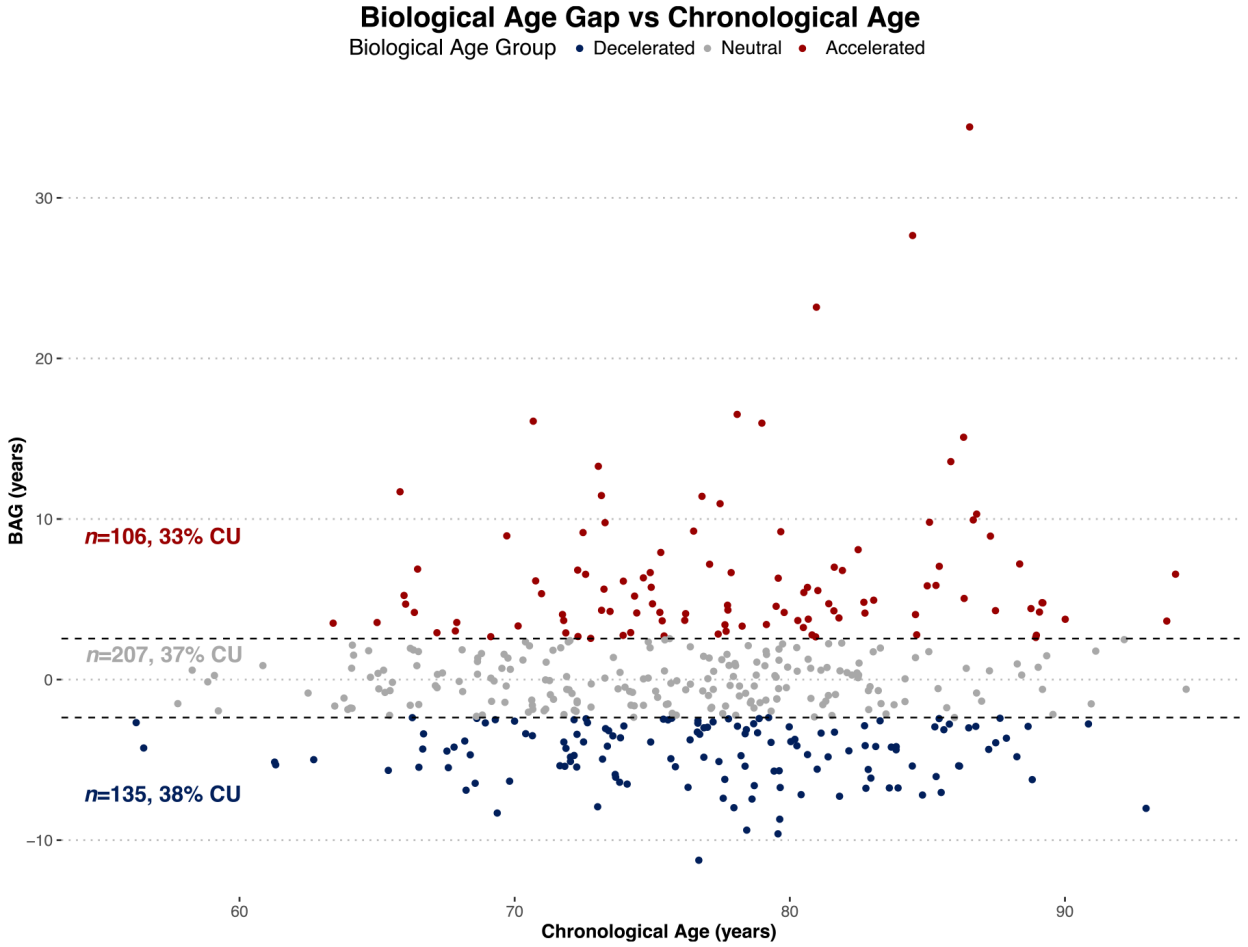
Classification of participants by biological age group. Participants with DNA methylation (*n* = 448) were classified into biological age groups based on their biological age gap averaged across clocks (BAG). Scatterplot of BAG versus chronological age, colored by biological age group, with a solid linear regression line in black and dashed lines showing the + /− 0.5 SD cutoffs: Accelerated (top, red) Decelerated (bottom, blue), Neutral (center, green). Sample sizes and % CU for each group are provided in text. There is no correlation between BAG and chronological age, suggesting that BAG likely captures additional variance encapsulating biological aspects of aging. BAG = Biological Age Gap; CU = Cognitively Unimpaired; SD = Standard Deviation.

Participants with DNA methylation data were stratified into biological age groups (accelerated, decelerated, and neutral) based on BAG as follows (see Fig 3). Accelerated: BAG was at least 0.5 standard deviation (SD) above the mean; Decelerated: BAG was at least 0.5 SD below the mean; Neutral: BAG was within 0.5 SD of the mean. The threshold of 0.5 SD, as opposed to a more extreme cutoff, was chosen to optimize the sample and effect sizes between the accelerated and decelerated groups.

All participants, regardless of DNA methylation availability, were grouped by chronological age to replicate results from literature (Dickerson et al., 2017) and serve as a reference for comparison: Younger (65 years and younger); Older (80 years and older); Average (between 65 and 80 years).

### 2.3. Neuroimaging measures of cortical structures

The participants in this study were enrolled across multiple phases of ADNI, namely ADNI1 and ADNIGO/2. Structural T1-weighted MRI acquired at 1.5 Tesla (T) or 3 T through ADNI (Mayo Foundation for Medical Education and Research., 2018) were processed and quantified using the *FreeSurfer* 5.1 cross-sectional pipeline (Fischl et al., 2004) to yield regional cortical thickness measures and volumes. Statistical harmonization methods were necessary to account for scanner differences affecting acquisition and to remove associated batch effects. The ComBat family of harmonization methods is frequently used to correct for batch effects in neuroimaging data (Fortin et al., 2018). ComBat uses Empirical Bayes models to harmonize means and variances of neuroimaging measures across batches. We applied ComBat to harmonize cortical thickness and volume measures at the ROI level using the *neuroHarmonize* package (Pomponio et al., 2020) in Python version 3.10.4. The batch variable was defined as the magnetic field strength of the scanner (either 1.5 T or 3 T). Prior and observed distributions by batch are illustrated in Figure S3. As established by prior studies (Fortin et al., 2018; Pomponio et al., 2020), the harmonization model was fit to all CU individuals and applied to symptomatic individuals. The effects of biological covariates, namely age, sex, and diagnosis, were preserved. Boxplots of cortical thickness and volume before and after harmonization in CU individuals are shown in Figure S3.

#### 2.3.1. Cortico-Medial Temporal index (CoMeT)

To quantify the relative involvement of cortex to MTL as a continuous measure, we defined the **Co**rtico-**Me**dial **T**emporal index (CoMeT) as follows. First, we identified brain regions of interest (ROIs) by approximately mapping neuroimaging signatures of AD (Bakkour et al., 2009; Dickerson et al., 2009; Möller et al., 2013; Touroutoglou et al., 2023) to the neuroanatomically-defined ROIs in the Desikan-Killiany-Tourville atlas (Desikan et al., 2006). Composite structural measures for cortex and MTL were computed by averaging bilateral ROIs across the respective brain areas (see Fig 1 for the list of ROIs). We then calculated w-scores by adjusting composite structural measures for age and sex (or age and intracranial volume for volumetric measures) using the full CU cohort as reference (see Table 1). Finally, CoMeT was calculated by subtracting the MTL composite w-score from the cortical composite w-score. We define the “cortical” composite as comprising all non-MTL regions, aligning with established literature discussing the “cortico-limbic” distribution of pathology (Murray et al., 2011; Kouri et al., 2024) i.e., cortical-predominant versus limbic-predominant or MTL-predominant presentations.

### 2.4. Statistical analysis

To study the association between CoMeT and BAG as continuous traits, we performed a correlation analysis using Pearson’s correlation coefficient. Wilcoxon rank-sum tests were used to statistically compare CoMeT across symptomatic individuals with decelerated or accelerated BAGs as well as younger and older symptomatic individuals. Effect sizes were computed as rank-biserial correlations and interpreted using established guidelines (Cohen, 1988, 1992). Chi-squared tests were conducted to assess the association between biological age groups and APOE4 alleles. Since all measures were adjusted for chronological age and sex, we do not additionally covary for chronological age or sex in our statistical analyses. All analyses were conducted in R version 4.4.0 using R Studio.

## 3. Results

### 3.1. Study population characteristics

Cross-sectional characteristics of the study participants by cohort are shown in Table 1. The CU and symptomatic cohorts were comparable for age, sex, and education level. We observed median CDRSB values of 0 for CU and 3 for symptomatic individuals. Among symptomatic individuals with MCI or dementia due to AD, the median CDRSB scores were slightly higher (1.5 and 5, respectively). The time between DNA methylation and the nearest MRI was less than 1 year on average among participants with both measures. Symptomatic individuals showed a higher frequency of carrying at least one APOE4 allele (68.5 %) compared to CU individuals (21.0 %). Chi-squared tests showed no significant differences by biological age groups across individuals with 0, 1, or 2 APOE4 alleles among symptomatic individuals, although these differences were significant in CU individuals (*χ*^2^=11.521, *p* = 0.021) (see Supplementary Table S1).

### 3.2. Validation of CoMeT in symptomatic individuals grouped by chronological age

**Fig. 4.**
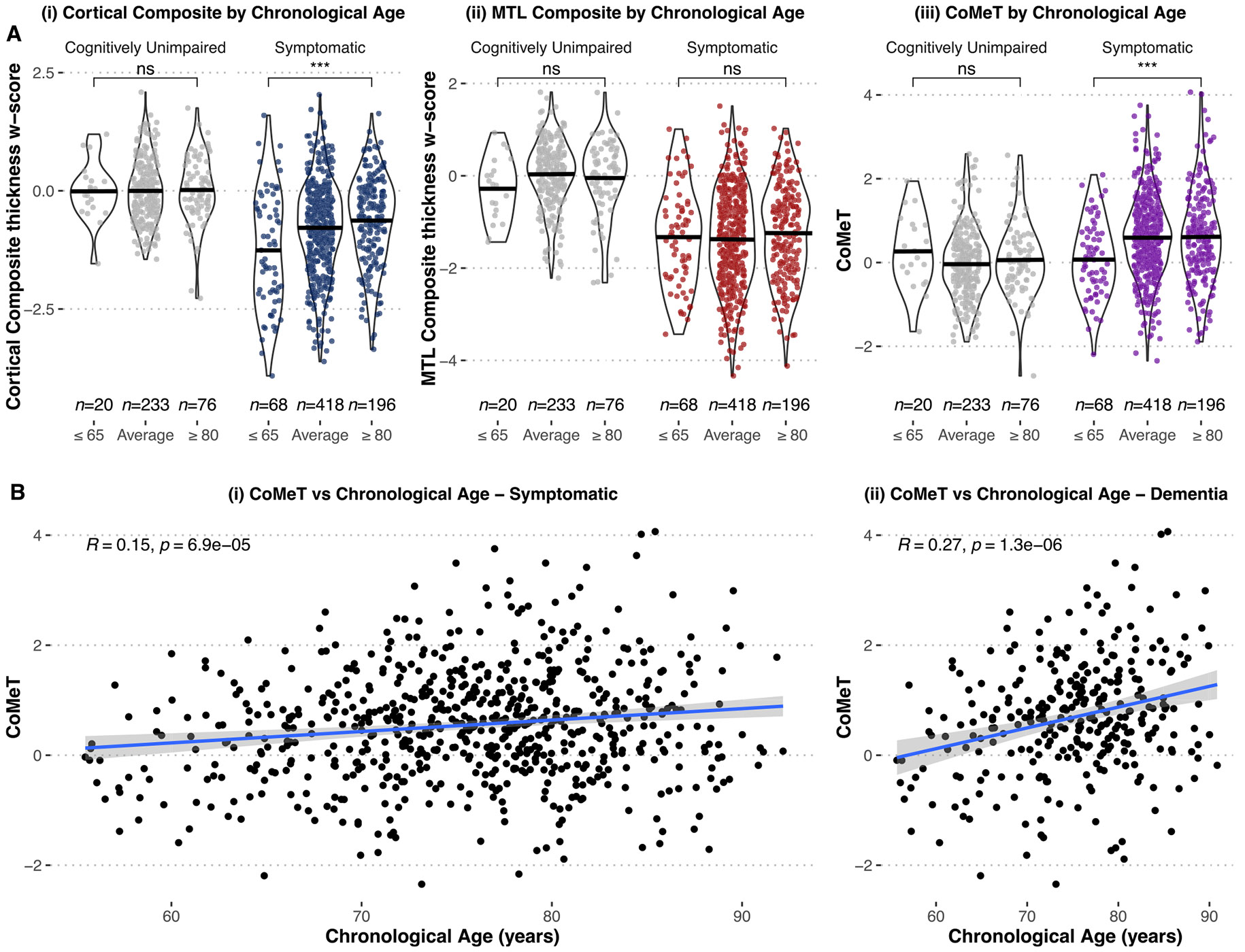
Association of chronological age with CoMeT. (A) Violin plots of (i) cortical composite thickness (ii) MTL composite thickness (iii) CoMeT, all adjusted for age and sex, by chronological age groups (younger, ≤ 65 years and older, ≥ 80 years) in CU individuals (left, grey) and symptomatic individuals (right, in color). the black crossbars show the mean of each distribution. In all comparisons, CU individuals are approximately centered at a zero mean after adjusting for age and sex to remove confounding effects due to healthy aging. Overall, symptomatic individuals show lower thickness in both cortex and MTL than CU individuals (*p* < 0.001). Younger symptomatic individuals shower significantly lower cortical thickness but no difference in MTL thickness compared to older symptomatic individuals. Critically, younger symptomatic individuals show significantly lower CoMeT scores than older symptomatic individuals (*p* < 0.001), indicating greater cortical involvement relative to MTL. (B) Scatterplots of CoMeT versus chronological age in (i) all symptomatic individuals, i.e., including ages 65–80 (Pearson *R*=0.15, *p* < 0.001) and (ii) dementia subgroup (Pearson *R*=0.27, *p* < 0.001) with linear regresson lines in blue. CU = Cognitively Unimpaired; CoMeT = Cortico-Medial Temporal index; MTL = Medial Temporal Lobe.

We first explored how CoMeT associates with chronological age and whether our results aligned with established findings (see Fig 4). Overall, CU individuals had mean structural w-scores of 0 in cortex and MTL as expected given that these measures were derived from the CU cohort. Symptomatic individuals had significantly atrophied structures compared to CU individuals in both cortex and the MTL (*p* < 0.001). There was a significant correlation between CoMeT and chronological age among all symptomatic individuals, including participants in the “average” group between 65 and 80 years of age (*n* = 682, Pearson *R*=0.15, *p* < 0.001). Among individuals with dementia only, this correlation was quantitatively stronger (*n* = 304, Pearson *R*=0.27, *p* < 0.001). Categorical analyses of chronological age groups revealed that younger symptomatic individuals had significantly lower cortical thickness compared to older symptomatic individuals with a medium effect size (*W*=4598, *p* < 0.001, rank-biserial correlation= −0.31) but no significant difference in MTL structure (*W*=6235, *p* = 0.430, rank-biserial correlation= −0.06). This emphasizes the importance of the relative difference (CoMeT) as opposed to cortical or MTL structures individually. As expected, CoMeT was significantly lower in younger individuals compared to older individuals within the symptomatic cohort with a small effect size (*W*=4751, *p* < 0.001, rank-biserial correlation= −0.29), reflecting greater cortical neurodegeneration relative to MTL in younger individuals. There were no associations between chronological age and CoMeT in the CU or MCI cohorts (see Figure S4).

### 3.3. Association of CoMeT with biological aging in the symptomatic cohort

**Fig. 5.**
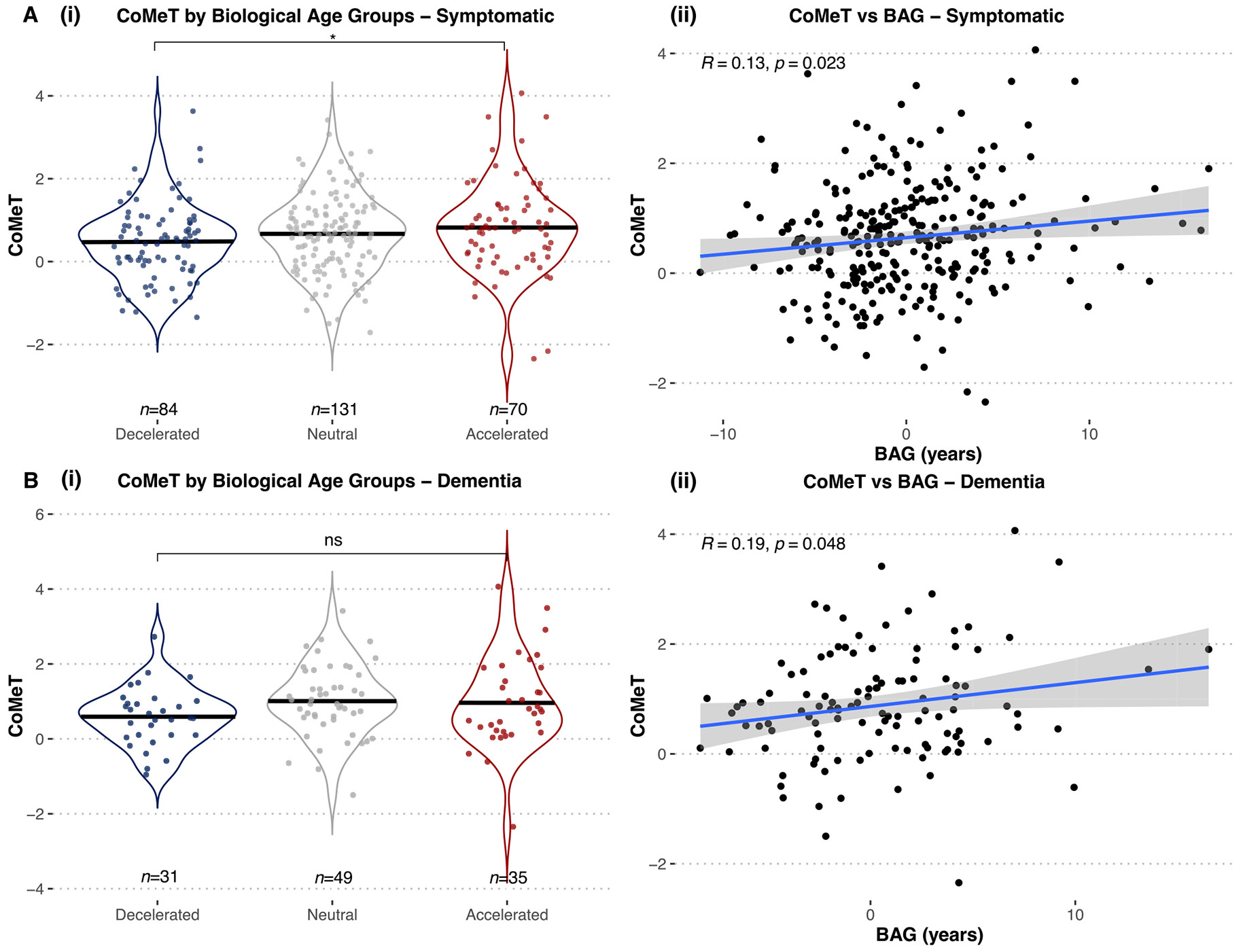
Association of BAG with CoMeT. Violin plots of CoMeT by biological age groups in (A, i) all symptomatic individuals and (B, i) the dementia-only subset. Symptomatic individuals with decelerated BAG have significantly lower CoMeT scores than those with accelerated BAG (*W*=2314, *p* = 0.023, rank-biserial correlation= −0.98). this difference is not significant when assessing individuals with dementia only, likely due to power constraints, but maintains a large effect size (*W*=445, *p* = 0.214, *R*ank-biserial correlation= −0.98). Scatterplots of CoMeT versus BAG with linear regression lines in blue in (A, ii) all symptomatic individuals (Pearson *R*=0.13, *p* = 0.023) (B, ii) dementia-only subset (Pearson *R*=0.19, *p* = 0.048). AD = Alzheimer’s disease; BAG = Biological Age Gap; CoMeT = Cortico-Medial Temporal index.

Analyses using continuous measures revealed a positive correlation between BAG and CoMeT (*n* = 285, Pearson *R*=0.13, *p* = 0.023), consistent with deceleration relating to relatively greater cortical involvement and acceleration relating to greater MTL involvement (see Fig 5). Categorical analyses revealed that CoMeT was significantly lower in individuals with decelerated BAG compared to individuals with accelerated BAG in the symptomatic cohort, with a large effect size (*W*=2314, *p* = 0.023, rank-biserial correlation= −0.98). Therefore, individuals with decelerated BAG show a less typical, more cortical-predominant disease pattern compared to individuals with accelerated BAG. CoMeT scores did not differ between individuals with accelerated or decelerated BAG in the CU or MCI cohorts (Figure S4).

We additionally performed a subgroup analysis of symptomatic individuals with dementia only, i.e., excluding MCI (see Fig 5) that also revealed a significant correlation between CoMeT and BAG (*n* = 115, Pearson *R*=0.19, *p* = 0.048), indicating relatively greater cortical involvement in individuals with decelerated BAG compared to individuals with accelerated BAG. While CoMeT scores were numerically lower in individuals with decelerated BAG compared to individuals with accelerated BAG, this result was not significantly different despite a large effect size, likely due to lower power and smaller sample sizes (*W*=445, *p* = 0.214, rank-biserial correlation= −0.98).

## 4. Discussion

Our results support established findings reporting an association between younger age of onset and disproportionately more cortical presentations of AD (Dickerson et al., 2017). As hypothesized, our results also demonstrate a significant association between biological aging and the spectrum of cortical to MTL atrophy in AD. Specifically, symptomatic individuals with decelerated BAGs exhibit significantly lower CoMeT scores, reflecting greater cortical involvement, compared to those with accelerated BAGs. Moreover, the mean CoMeT score of symptomatic individuals with “neutral” BAGs fell in between the means of their decelerated and accelerated counterparts. This suggests that the relationship between biological aging and atypical neurodegenerative pattern exists across a continuum. Indeed, CoMeT and BAG correlate when examined across all symptomatic individuals. This association appears at least quantitatively stronger when limited to individuals with dementia due to AD (i.e., excluding those with MCI due to AD). We observe no significant association in the MCI subgroup, suggesting that the manifestations of biological age on atypicality may emerge more prominently with later disease progression. It is also worth noting that since the neutral group represents the largest proportion of individuals and has CoMeT scores between the two extreme groups, one might also consider the biologically accelerated group as having a more limbic-predominant “atypical” presentation, as described in the literature, while the biologically decelerated group has more cortical-predominant, atypical cases (Murray et al., 2011; Ferreira et al., 2020).

These results align with the effects of chronological age on disease presentation as frequently noted in prior reports (Swerdlow, 2007; Dickerson et al., 2017) and suggest that biological age captures additional variance. Therefore, biological age may be a core factor modulating the topography of AD pathology and may further explain some cases of discordance between chronological age and degree of atypicality, for instance, older-onset cases with more cortical involvement.

Biological aging may also interact with other factors to influence AD presentation. Given that MTL tau deposition is commonly associated with healthy aging (Braak and Braak, 1991), older adults may already exhibit an MTL-localized epicenter driving the clinical manifestations of AD. This predisposition could result in a more limbic-predominant presentation as the disease progresses. As our findings suggest, individuals with accelerated BAG tend to present with higher CoMeT scores, indicating greater MTL involvement.

As with patterns of atrophy, neuropathological studies have also consistently shown that the distribution of tau pathology is not uniform across patients (Arezoumandan et al., 2022). While most individuals exhibit a mix of MTL and cortical involvement, some exhibit more “atypical” burdens with disproportionately heavy burden isolated in the MTL or in cortical regions (Murray et al., 2011; Kouri et al., 2024). These patterns are often linked to differences in clinical symptoms, age of onset, and the presence of co-pathologies (Ossenkoppele et al., 2016). For example, AD cases with more cortical tau accumulation tend to exhibit earlier disease onset and atypical cognitive symptoms, such as language or visuospatial deficits, rather than the more classic memory impairments seen with MTL-centered pathology (Frontzkowski et al., 2022). Some studies have suggested that certain genetic factors, such as APOE4 genotype, might influence whether tau pathology predominantly affects the cortex or the MTL (Therriault et al., 2020; Steward et al., 2023).

The corticolimbic index (CLix) (Kouri et al., 2024) is a recently developed neuropathological measure derived from *ex vivo* brain tissue analysis that quantifies the relative burden of tau pathology in the cortex versus the MTL. CLix was developed to explore corticolimbic vulnerability, conceptualizing tau pathology as a continuous trait that varies across individuals, thus providing a spectrum of disease topography from predominantly cortical involvement to predominantly MTL involvement. CoMeT is analogous to CLix as a continuous measure that, in contrast, can be assessed with structural MRI during life. Furthermore, CoMeT can be used in conjunction with other *in vivo* biomarkers, including amyloid- and tau-PET, CSF, plasma, and multiomics data to explore how different factors contribute to the corticolimbic distribution of atrophy. This allows for the dynamic tracking of pathology and its clinical correlates over time, facilitating future longitudinal studies that could elucidate the progression of AD and its variants.

Recent studies have highlighted that early-onset Alzheimer’s disease (AD), often clinically labeled as “amnestic”, may manifest with non-amnestic, dysexecutive presentations more frequently than previously recognized (Townley et al., 2020; Corriveau-Lecavalier et al., 2024; Putcha et al., 2025). Notably, analyses of the LEADS cohort demonstrated that a significant proportion of individuals under 65 diagnosed with “amnestic AD” exhibited prominent dysexecutive symptoms rather than memory impairment (Putcha et al., 2025). Our findings of greater cortical relative to MTL involvement in individuals with decelerated biological aging may align with these emerging clinical observations, suggesting that some early-onset, MTL-sparing patients within the ADNI dataset could indeed reflect a dysexecutive phenotype. This highlights the importance of considering biological aging in clinical phenotypic variability, especially in the context of structural neuroimaging findings in AD. While this paper focuses on pathological and neurodegenerative heterogeneity in this paper, future research could expand on our work by studying associations between biological aging and heterogeneity in clinical presentation.

There are a few limitations in this study that may also be overcome through future research. Firstly, we utilized DNA methylation data from blood rather than brain tissue. Since epigenetic modifiers of gene expression are tissue specific, it would be ideal to study methylation from the cortex and MTL to understand these age effects more directly. However, this type of data is very rare and previous studies have shown a high correlation among predicted outcomes from clocks trained on multiple tissues and applied to blood methylation data (Horvath, 2013; Shireby et al., 2020). Moreover, blood methylation allows for *in vivo* estimation of biological age, as opposed to brain tissue which would necessitate *ex vivo* analysis. Secondly, the study population had limited representation of individuals under 65 years of age and was constrained due to the incorporation of multi-modal data including DNA methylation, imaging, and amyloid status. Lastly, our sample was primarily comprised of highly educated participants who self-identified as White. This homogeneity may affect the generalizability of these results and is crucial to address for broader applicability. Future work is therefore necessary to evaluate the generalizability of these findings and the application of biological aging measures in more diverse datasets across the life-course. Additionally, development of new epigenetic clocks tailored to aging populations and accounting for co-pathologies may better inform our understanding of age-related disease trajectories.

This is the first study, to our knowledge, investigating the role of biological aging in driving heterogeneity in the neuroanatomic distribution of AD. We define a novel continuous measure, CoMeT, to operationalize and quantify the spectrum of neurodegenerative patterns. As hypothesized, we observed that decelerated BAG is associated with a less typical disease presentation with relatively greater cortical involvement, while accelerated BAG is linked to relatively greater MTL involvement. Future studies involving pathway and enrichment analyses may identify specific epigenetic modifiers and genetic variants driving this heterogeneity.

We conclude that biological aging modulates atypical disease presentations in AD in conjunction with and beyond chronological age. This study suggests that fundamental effects of the aging process contribute to the neuroanatomic presentation of AD, and our work contributes to a better understanding of the biological basis and mechanisms underlying AD heterogeneity.

## Supplementary Material

1

## Figures and Tables

**Table 1:** Characteristics of the cross-sectional study population. Demographics and clinical information of the cross-sectional study participants by cohort. All participants have MRI and amyloid status. The “Cognitively Unimpaired” (CU) cohort includes all amyloid-negative, CU individuals. The “Symptomatic” cohort includes all amyloid-positive individuals with either MCI or dementia due to AD. The interval between MRI and DNAm as well as frequencies by biological age groups are provided for the subset of participants with DNA methylation (448 total, *n* = 163 for CU, *n* = 285 for symptomatic). All values are in specified units in mean (SD) format or as percentages except for CDRSB and education, which are in median (IQR) format.

	Cognitively Unimpaired	Symptomatic
**N**	329 (163 with DNAm)	682 (285 with DNAm)
**Clinical Diagnosis (N)**	-	MCI (378, 170 with DNAm) Dementia (304, 115 with DNAm)
**Age at MRI** (years)	74.84 (6.96)	75.57 (7.43)
**Sex** (% female)	50.8 %	41.3 %
**APOE4** (% carrier)	21.0 %	68.5 %
**CDRSB**	0 (0)	3 (3.5)
**Education** (years)	16 (3)	16 (4)
**BAG** (years)	0.21 (5.69)	0.03 (4.42)
**MRI to DNAm(years)**	−0.98 (1.55)	−0.64 (1.36)
**Biological Age Groups** (%)	Accelerated (22.1 %), Neutral (46.6 %), Decelerated (31.3 %)	Accelerated (24.6 %), Neutral (46.0 %), Decelerated (29.5 %)

Abbreviations: AD = Alzheimer’s disease. APOE4 = Apolipoprotein E4 allele. BAG = Biological Age Gap. CDRSB = Clinical Dementia Rating scale Sum of Boxes. CU = Cognitively Unimpaired. DNAm = DNA methylation. IQR = Inter Quartile Range. MCI = Mild Cognitive Impairment. MRI = Magnetic Resonance Imaging. SD = Standard Deviation.

## Data Availability

The ADNI data is shared without embargo through the Laboratory of Neuroimaging (LONI) Image and Data Archive (https://ida.loni.usc.edu/login.jsp), a secure research data repository. Scientists may obtain access to imaging, clinical, genomic (including DNA methylation), and biomarker data for the purposes of scientific investigation, teaching, or planning clinical research studies via application. Access is contingent on adherence to the ADNI Data Use Agreement and the publication policies. More information is available online at https://adni.loni.usc.edu/data-samples/access-data/. In addition, the analysis code and documentation will be made available publicly after the manuscript is accepted.

## Appendix A. Supporting information

Supplementary data associated with this article can be found in the online version at doi:10.1016/j.neurobiolaging.2025.06.003.
