# Supplementary material for: Biological age acceleration in Alzheimer’s disease modulates relative cortical to medial temporal lobe neurodegeneration
Source: Neurobiol Aging. Author manuscript; Available in PMC 2026 Apr 17. (PMC13089110; doi:10.1016/j.neurobiolaging.2025.06.003)
Supplement: 1 [file NIHMS2162017-supplement-1.docx]

**Figure S1. Quality control of processed DNA methylation data and UMAP visualization.**

DNA methylation was assayed using the Illumina Infinium Human Methylation EPIC v1.0 BeadChip. Raw intensity files were processed using the *SeSAMe* R package according to standard protocols, including sample filtering, CpG probe filtering, signal correction, and batch normalization. Quality control procedures assessed the signal detection frequency and sample separation by covariates. **(A)** Density plot of the signal detection frequency across all CpGs, which was 95.8% on average. **(B)** Scatterplots of Uniform Manifold Approximation and Projection (UMAP) dimensions 1 and 2 by covariates (sex, array, plate, clinical diagnosis), including all participants with DNA methylation (*n*=448). Samples separate by sex but not significantly by other factors. Therefore, sex was included as a covariate in downstream analyses. CpG = cytosine-guanine dinucleotide.

**Figure S2. Covariance heatmap of epigenetic clock measures.**

Correlation matrices including all study participants with DNA methylation data (*n*=448) illustrating the high covariance among epigenetic clocks. The upper triangle is a heatmap with the Pearson R value and significance level (*** = *p* < 0.001) between each pair of age measures. Lighter red indicates lower correlation, approaching 0, and darker red indicates higher correlation, approaching +1. The lower triangle shows a scatterplot of each pair of age measures with a linear regression line in blue. Density plots of each measure are along the diagonal. **(A)** Correlation matrix of chronological age; epigenetic clock ages from three clocks: Horvath 2013, Hannum 2013, and Shireby 2020; and the average clock age. All correlations are significant. The residuals of each clock age regression against chronological age (first column) are the clock BAGs. **(B)** Correlation matrix of chronological age, clock BAGs, and the average clock BAG. There is no correlation between BAG and chronological age. Clock BAGs correlated modestly with each other and highly with the average. BAG = Biological Age Gap.

**Figure S3. Harmonization of Neuroimaging Thickness Measures*.***

Cross-sectional thickness measures derived from MRI were harmonized to adjust for batch effects. The batch variable was defined as scanner field strength, which was either 1.5 Tesla (T) or 3T. The ComBat harmonization model (*neuroHarmonize* Python package) was fit to all CU individuals, preserving the effects of age and sex. The fitted model was then applied to symptomatic individuals. **(A)** Density plots of prior (dashed) and observed (solid) distributions of batch effects for each batch, i.e., scanner field strength, with 1.5T shown in orange and 3T shown in brown. **(B)** Violin plots of left hippocampal volume (**i** and **ii**) and thickness measures in left precuneus (**iii** and **iv**), which is one of the cortical signature brain regions in AD. Plots show all CU individuals (*n*=329). Blue crossbars indicate the means of each distribution. Raw thickness measures (left, **i** and **iii**) are significantly different across batches, while harmonized thickness measures (right, **ii** and **iv**) are no longer significantly different across batches. AD = Alzheimer’s disease. CU = Cognitively Unimpaired.

**Table S1. Chi-squared analysis of biological age groups by APOE4 alleles.**

A chi-squared test of biological age groups (decelerated, neutral, and accelerated) by the number of APOE4 alleles (0, 1, or 2) in **(A)** all CU individuals with DNA methylation (*n*=163) revealed a significant association between the two variables and **(B)** all symptomatic individuals with DNA methylation (*n*=285) revealed no significant association between the two variables. The frequency tables and chi-squared statistics are below. CU = Cognitively Unimpaired.

**(A) Pearson’s Chi-squared test:**

*X*-squared=11.521, df=4, *p*-value=0.021

|  | **0** | **1** | **2** |
| --- | --- | --- | --- |
| **Decelerated** | 48 | 3 | 0 |
| **Neutral** | 54 | 20 | 2 |
| **Accelerated** | 30 | 6 | 0 |

**(B) Pearson's Chi-squared test:**

*X*-squared=1.9665, df=4, *p*-value=0.742

|  | **0** | **1** | **2** |
| --- | --- | --- | --- |
| **Decelerated** | 30 | 41 | 13 |
| **Neutral** | 42 | 66 | 23 |
| **Accelerated** | 22 | 40 | 8 |

**Figure S4.** **Association of CoMeT with Chronological Age and BAG in CU and MCI individuals**

Scatterplots of CoMeT in **(A)** CU individuals and **(B)** MCI individuals by **(i)** chronological age (*n*=329, CU and *n*=378, MCI) and **(ii)** BAG (*n*=163, CU and *n*=170, MCI), with blue regression lines overlaid. CoMeT was not significantly correlated with chronological age or BAG in neither CU nor MCI individuals. BAG = Biological Age Gap. CU = Cognitively Unimpaired. MCI = Mild Cognitive Impairment.
